# Composition and Biodiversity of Culturable Endophytic Fungi in the Roots of Alpine Medicinal Plants in Xinjiang, China

**DOI:** 10.3390/jof11020113

**Published:** 2025-02-03

**Authors:** Mengyan Hou, Jun Zhu, Chunyan Leng, Xinjie Huang, Mingshu Yang, Yifei Yin, Yongmei Xing, Juan Chen

**Affiliations:** 1 State Key Laboratory for Quality Ensurance and Sustainable Use of Dao-di Herbs, Institute of Medicinal Plant Development, Chinese Academy of Medical Sciences & Peking Union Medical College, No.151, Malianwa North Road, Haidian District, Beijing 100193, China; houmy8217@163.com (M.H.); lcy445089@163.com (C.L.); 17830667856@163.com (X.H.); yangmingshugm@163.com (M.Y.); yinyifei0226@163.com (Y.Y.); 2 Xinjiang Institute of Chinese and Ethnic Medicine, Urumqi 830002, China; zhujun1hao@163.com; 3 School of Pharmacy, Shandong Second Medical University, Weifang 261053, China

**Keywords:** alpine plant, endophytic fungi, DSE fungi, fungi diversity

## Abstract

(1) Background: Endophytic fungi play an important role in plant growth and stress resistance. The presence of a special fungal taxon such as the dark septate endophytic (DSE) fungi in alpine environments is particularly important for plant resistance to environmental stresses. However, the composition of root endophytic fungi in different environments and between different host plants has not been well studied. (2) Results: A total of 408 culturable endophytic fungi were isolated from the roots of *Saussurea involucrata* and *Rhodiola crenulata* which were collected in 5 plots from the Tianshan and Karakoram Mountains of the Xinjiang region, belonging to 91 species, 54 genera, 31 families, and 3 phyla based on the morphological characteristics and molecular sequence. Among them, DSE fungi were the dominant group, accounting for 52.94%, and *Leptodontidium orchidicola* was the dominant species. In addition, we also compared the composition and diversity of root endophytic fungi from different plants and different sites, with emphasis on special fungal taxa such as DSE. (3) Conclusions: The composition and diversity of cultural endophytic fungi are significantly different in the two alpine medicinal plant species and across various locations. Some fungi showed the preferences of the host or environment. The endophytic fungal resources, especially DSE, were very rich in the two alpine medicinal plants, indicating that these fungi may play a crucial role in the ecological adaptation of host plants in harsh environments.

## 1. Introduction

Microbial communities respond to changing environmental conditions by regulating global biogeochemical cycles such as plant nutrient uptake and soil carbon storage [1,2]. As global climate change progresses, biodiversity is declining [3]. Alpine ecosystems face even greater challenges, as a climate shift occurs there at twice the global average rate [2]. Rising temperatures drive alpine plants to migrate to higher elevations, placing many of them at risk of “summit extinction” [4]. Mountains, covering 25% of the Earth’s land surface, are rich in biodiversity due to their complex climates and topographies. Compared to low-altitude regions, alpine areas are characterized by high elevations, hypoxia, large diurnal temperature variations, and intense radiation [5]. The mountains of Xinjiang, China, support diverse vegetation shaped by geological uplift, glaciation, and monsoonal changes, where plants often contend with various environmental challenges [6].

Xinjiang is located in the northwest of China, with a vast territory, including the Tianshan, Karakoram, Altai Mountains, and the Junggar and Tarim basins [7]. The unique topography and climate of Xinjiang region breed abundant plant resources, including valuable medicinal species like *Saussurea involucrata*, *Rhodiola crenulata*, and *Lithospermum* [8,9]. *Saussurea involucrata* (Kar. & Kir.) Sch. Bip. belonging to the Asteraceae family, is a Chinese traditional medicinal plant for regulating meridians and promoting blood circulation [10]. Pharmacological studies have shown that *S. involucrata* oral liquid (SIOL) can alleviate rheumatoid arthritis in clinical applications [11], while *S. involucrata* polysaccharide (SIP) demonstrates significant antioxidant and photoprotective effects on skin exposed to UVB radiation [12]. *S. involucrata* is primarily distributed in Xinjiang, Russia, and Kazakhstan, growing at altitudes of 2400–4100 m [13]. *Rhodiola crenulata* (Hook. f. & Thomson) H. Ohba is also a famous medicinal plant in the Crassulaceae family, which shares a similar habitat with *S. involucrata* and grows at altitudes of approximately 2800–5600 m, and the two species are often found coexisting [14]. According to the Chinese Pharmacopoeia, *R. crenulata* is used for relieving cough, strengthening the spleen, and calming the mind. Pharmacological studies have shown that *R. crenulata* is used to treat cardiovascular diseases, tumors, and diabetes [15]. However, due to the climate changes, over-harvesting of wild resources, and the lack of large-scale cultivation, both species are now on the brink of extinction and are listed as second-class protected wild plants in the National List of Key Protected Wild Plants [16].

In recent years, with the advancement of sequencing technology, plants have increasingly been considered as complex assemblies of plants and their associated microorganisms, of which endophytic fungi are important components [17,18]. Endophytic fungi ubiquitously colonize the internal tissues of host plants for part or all of their lifecycle without inducing apparent disease symptoms [19]. Studies have shown that the endophytic fungi can promote plant growth and development, assist in resisting environmental stresses, and facilitate the production of secondary metabolites [20]. Among the endophytic fungi, dark septate endophytic (DSE) fungi form a crucial subgroup. DSE refers to fungi that colonize plant roots with dark, septate hyphae, without causing noticeable symptoms of disease [21]. Previous studies have indicated that DSE fungi frequently colonize plants in unique environments and contribute to host plant resilience against harsh conditions, as well as promoting plant growth [22,23]. The ability of *S. involucrata* and *R. crenulata* to grow in such harsh environments may be closely related to their microbial communities, particularly DSE.

Moreover, current research on these two alpine medicinal plants primarily focuses on morphology, chemical composition, and pharmacology [24,25]. Limited studies on their endophytic fungi, especially those on the composition of DSE, the most predominant group, are unknown. In light of this, the study identifies culturable endophytic fungi from the roots of two alpine medicinal plants collected from 5 sampling sites in the Tianshan and Karakoram Mountains in Xinjiang. The study aims to understand the diversity and distribution patterns of the culturable endophytic fungi, particularly DSE, in these plants, and to lay the foundation for further analysis of the symbiotic relationship between alpine plants and fungi. At the same time, this study also aims to acquire valuable culturable endophytic fungi from these rare alpine medicinal plants, which may provide precious fungal resources for future artificial cultivation and conservation of these endangered medicinal plants, as well as the preservation of alpine biodiversity in the future by the microbiological technique.

## 2. Materials and Methods

### 2.1. Sample Collection

Xinjiang, the largest province in China, is located in the northwest (73°40′ E–96°18′ E, 34°25′ N–48°10′ N). This vast region features diverse landscapes, including mountains, basins, and deserts, with unique geomorphic and ecological characteristics. Two alpine medicinal plants, *S. involucrata* (Asteraceae) and *R. crenulata* (Crassulaceae), were selected from the Tianshan and Karakorum Mountains, two major mountain ranges in Xinjiang. The Tianshan Mountains have a temperate continental arid climate, whereas the Karakorum Mountains are situated in a transitional zone between the Qinghai-Tibet alpine region and the northwest arid region.

Root and soil samples from two alpine plants were collected from 5 sites across the Tianshan and Karakoram Mountains in Xinjiang, with their original habitat, latitude, longitude, and altitude recorded (Table 1). At each sampling site, 3 large plots (10 m × 10 m) with similar terrain were selected, each plot spaced approximately 100 m apart. Within each plot, 3 to 5 healthy plants were randomly selected, ensuring a minimum spacing of 10 m between individual plants. All the samples were collected and stored at 4 °C, and transported under low-temperature conditions to the laboratory.

### 2.2. Root Sample Processing, Morphological and Anatomical Observation

The root samples were washed under running water and stained using Trypan blue (Solarbio, Beijing, China) according to the steps for observing mycorrhizal fungi [26]. In brief, following the material processing method described by Phillips and Hayman (1970), the roots were cut into 1 cm segments and fixed in Formalin-Aceto-Alcohol (FAA) (Coolaber, Beijing, China) solution for 24 h, then washed with running water. The root segments were transferred to 10% KOH (Beilian, Tianjin, China) and heated in a 90 °C water bath for 40–60 min until the roots became transparent, removing the cytoplasm from the root epidermal cells. The washed root segments were immersed in a 5% lactic acid solution (XiLONG SCIENTIFIC, Shantou, China) for 5 min to facilitate staining. The acidified material was directly transferred to a 0.05% Trypan blue-lactic acid glycerol solution for 3 min of staining, then rinsed multiple times with glycerol (Yongda, Tianjin, China) and left to decolorize overnight. The root samples were placed on a glass slide and observed under the optical microscope (ZEISS Axio Imager A1, Jena, Germany), with images captured.

### 2.3. Isolation and Cultivation of Endophytic Fungi

Healthy, fresh, robust, and dark brown roots were selected and washed thoroughly with running water to remove soil and debris, and blotted dry. Root segments were soaked in 75% ethanol for 30 s, immersed in 5% NaClO (Beilian, Tianjin, China) for 3–5 min, rinsed with sterile water, and immersed in a 10 mL solution containing 150 µg/mL streptomycin sulfate and 150 µg/mL potassium penicillin (Gibco, Grand Island, NY, USA) for 10 min, and washed 3 times with sterile water. The solution from the last rinse was collected and 1 mL was placed on PDA agar plates, incubated at 25 °C in the dark for 3 days with 5 replicates. The absence of colony growth indicated effective sterilization of the root surfaces. The completely sterilized roots were cut into 2 mm sections and placed on 1 cm^3^ PDA agar blocks containing dual antibiotics (100 µg/mL streptomycin sulfate and 100 µg/mL potassium penicillin), resulting in a total of 4248 root segments. In addition, 9 PDA agar blocks were placed on each 9 cm Petri dish, with one root segment inoculated per block, resulting in a total of 472 dishes. The dishes were sealed and incubated at 25 °C in the dark for 7–10 days, with regular observations and timely treatment of contaminated plates. When colonies formed on the agar, the edges of actively growing hyphae were transferred to PDA agar for purification, and once pure colonies were obtained, they were subcultured onto PDA slants and stored in 10% glycerol tubes. The strains used in this study were stored in the strain collection of the Biotechnology Center of the Institute of Medicinal Plant Development, Chinese Academy of Medical Sciences.

### 2.4. Identification of Culturable Endophytic Fungi

The strains isolated were identified using both morphological observation and molecular biology techniques. Morphological identification was carried out by observing colony characteristics, such as shape, color, and exudates, as well as microscopic features like hyphae, spore-producing structures, and spores under a ZEISS Axio Imager A1 microscope (ZEISS, Jena, Germany). The identification was based on the latest fungal classification system.

Molecular identification was performed by grinding the mycelia in liquid nitrogen, followed by DNA extraction using the CTAB method. DNA concentration was measured using Nanodrop (Thermo NanoDrop 2000, Waltham, MA, USA) and PCR amplification of (internal transcript space), and ITS was performed with ITS1 and ITS4 primers. The PCR conditions were as follows: 95 °C for 5 min (initial denaturation), 94 °C for 1 min (denaturation), 52 °C for 50 s (annealing), 72 °C for 1 min (extension), followed by a final extension at 72 °C for 7 min, with 35 cycles [27]. The amplification products were analyzed using 1% agarose (Biowest, Nuaillé, France) gel electrophoresis (BIO-RAD, Hercules, CA, USA; Agilent 2100 Bioanalyzer, Santa Clara, CA, USA) to verify their quality, and the qualified PCR products were sent to GeneWiz (Suzhou, China) for sequencing. The sequence chromatograms were analyzed using SnapGene software (4.3.6), and after manual quality control, high-quality sequences were obtained and subjected to BLAST analysis on the NCBI website (https://blast.ncbi.nlm.nih.gov/Blast.cgi (accessed on 20 November 2024)). Combining the molecular data with morphological features, a comprehensive species identification was performed.

### 2.5. Data Analysis

Statistical analysis was performed to determine the isolation rate and relative frequency of endophytic fungi in each plant tissue. The isolation rate (IR) was used to calculate the percentage of root segments infected by endophytic fungi, indicating the degree of infection. The relative isolation frequency (IF) was used to calculate the percentage of each fungal species relative to the total number of culturable endophytic fungi, determining the species dominance. The Richness index refers to the total number of fungal species at that site. The Shannon–Wiener and Simpson diversity indices were used to assess the diversity of endophytic fungal communities [28,29]. The Pielou evenness index was used to measure the evenness of the fungal community distribution across the samples [30]. Sorenson’s similarity coefficient (Cs) was used to compare the similarity of endophytic fungal composition between the samples [31]. Cs range from 0 to 1, with values closer to 0 indicating lower similarity between the endophytic fungal communities of the two locations. When Cs equals 0, it indicates that the endophytic fungal communities of the two locations are completely different. The formulas for these calculations are as follows: The relative abundance is calculated by dividing the number of isolated strains of a particular taxon by the total number of isolated strains across all taxa. The isolation rate (IR) is calculated by dividing the total number of root segments infected by fungi by the total number of root segments cultured.

Shannon–Wiener index: H’ = −$\sum_{i-1}^{s}($P_i_)(lnP_i_), P_i_ = n_i_/N, N represents the total number of fungal species obtained from a particular site; n_i_ represents the number of individuals of the i-th fungal species at the site.

Simpson index: D = 1 − $\sum_{i-1}^{s}(P$_i_)^2^, P_i_^2^ = n_i_(n_i_ − 1)/N(N − 1), N is the total number of fungal species obtained from a specific site; n_i_ is the number of individuals of the i-th fungal species at that site.

Pielou index: J = H/lnS, S represents the total number of species at the site.

Sorenson’s similarity coefficient: Cs = 2j/a + b, j represents the number of fungal species common between different sites, while a and b represent the number of fungal species at each site, respectively.

## 3. Results

### 3.1. Comparison of Cultural Endophytic Fungal Community Structure and Diversity in Two Alpine Plants

**Figure 1:**
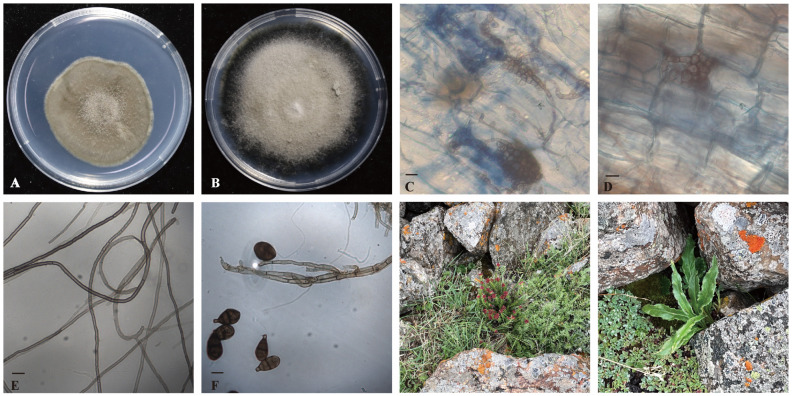
Observation of the sampling sites of two alpine medicinal plants and morphology of isolated fungi. (**A**,**B**) Typical images of isolated DSE fungi. (**C**,**D**) Microscopic observation of DSE colonized in alpine plant roots. (**E**,**F**) Microscopic view of DSE on PDA medium ((**E**) *Cadophora* sp., (**F**) *Alternaria alternata*). (**G**,**H**) Observations of sampling sites for two alpine plants ((**G**) *R. crenulata*, (**H**) *S. involucrata*). The scale bar in the legend represents 10 μm.

Fresh root segments of two alpine medicinal plants were fixed with FAA fixative and subsequently stained with trypan blue to observe the colonization of endophytic fungi. The results revealed substantial fungal colonization in the roots of both wild *S. involucrata* and *R. crenulata*. The colonizing fungal hyphae were brown and septate, forming bead-like structures within the cells. Based on the morphological characteristics of the hyphae, these fungi were identified as belonging to the typical DSE fungi (Figure 1).

A total of 4248 root tissue blocks from two alpine medicinal plants across 5 sampling sites were used for endophytic fungal isolation and 3356 blocks produced fungal colonies. Fungi with similar morphological characters were divided into a morphological group, and molecular identification was performed on 408 endophytic fungal isolates, which were classified into 3 phyla, 9 classes, 16 orders, 31 families, 54 genera, and 91 species (Table S1). Ascomycota was the dominant phylum, with a total of 392 isolates accounting for 96.08% of the total fungal count. Helotiales was the dominant order, with 264 isolates representing 64.71% of the total fungal count and *Leptodophora* was the most prevalent genus, with 64 isolates constituting 15.69% of the total fungal count.

**Figure 2:**
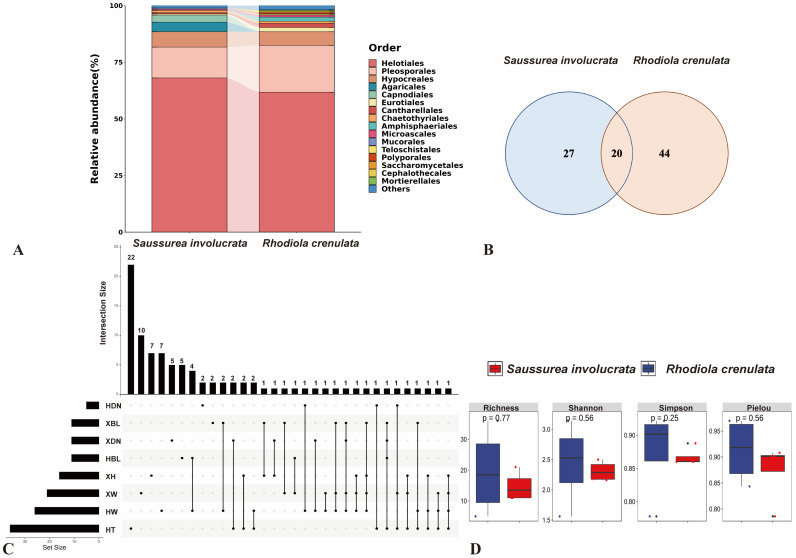
Comparison of culturable endophytic fungal communities between two alpine medicinal plants. (**A**) Structure of culturable endophytic fungal communities in two medicinal plants. (**B**) Venn diagram of the endophytic fungal community of two medicinal plants. (**C**) Venn diagram of culturable endophytic fungal communities of two alpine medicinal plants from different sites. XW, XBL, XDN, and XH were the sampling sites of *S. involucrata,* and HW, HBL, HDN, and HT were the sampling sites of *R. crenulata*. (**D**) Comparison of the diversity of culturable endophytic fungal communities in two medicinal plants.

The isolation rates, abundances, and community structures of culturable endophytic fungi from the two alpine plants exhibited significant differences (Figure 2A). A total of 191 isolates belonging to 47 species of endophytic fungi were obtained from the roots of *S. involucrata*, while 217 isolates belonging to 64 species were obtained from the roots of *R. crenulata*. The composition of endophytic fungal taxa from the two medicinal plants is significantly different, with 27 species unique to *S. involucrata* and 44 species unique to *R. crenulata*. *L. orchidicola* and *Filosporella* sp. were identified as the dominant endophytic fungi in the roots of *S. involucrata* and *R. crenulata*, respectively, with relative isolation frequencies of 20.42% and 9.68% in their corresponding host root segments. The different composition of the two plants indicates that the host plants selectively recruit different fungi to form their endophytic fungal communities. Furthermore, common characteristics were observed in the structural composition of the culturable endophytic fungal communities between the two medicinal plants, with 20 species distributed in both plants (Figure 2B), including *Phoma schachtii, Plenodomus meliloti, Alpinaria rhododendri, Alternaria alternata, Alternaria* sp., *Articulospora, Filosporella* sp., *L. orchidicola, Rhexocercosporidium* sp., *Tetracladium* sp., *Leptosphaeria* sp., *Cadophora* sp., *Mycochaetophora,* uncultured Helotiales, *Graphium basitruncatum*, *Dactylonectria macrodidyma*, *Neonectria candida*, *Neonectria* sp., *Thyridium cornearis*, *Monodictys arctica*, etc., resulting in a Cs similarity of 36.04%.

In three collection sites, Tianshan Mountain No.1 glacier (XW and HW), Bayinbuluke, Hejing County (XBL and HBL), and Danangou Uzbek Township (XDN and HDN), which are in the Tianshan Mountains (Table 1), both medicinal alpine plants were collected, with distinct differences in the composition of their root-associated endophytic fungal communities. In these collection sites, fungi isolated from 26 *S. involucrata* plants belonged to 3 phyla, 8 orders, and 16 families. Similarly, in the same 3 locations, fungi isolated from 26 *R. crenulata* plants belonged to 3 phyla, 8 orders, and 17 families. Nine families were shared between the two plants, including Melanommataceae, Didymellaceae, Helotiaceae, Leptodontidiaceae, Ploettnerulaceae, Aspergillaceae, Pleosporaceae, Nectriaceae, and Thyridiaceae. Seven families were unique to *S. involucrata*, including Ceratobasidiaceae, Cladosporiaceae, Phaeosphaeriaceae, Herpotrichiellaceae, Bionectriaceae, Hypocreaceae, and Mucoraceae. Eight families were unique to *R. crenulata*, including Hydnaceae, Discinellaceae, Mollisiaceae, Sclerotiniaceae, Saccharomycetaceae, Amphisphaeriaceae, Bartaliniaceae, and Mortierellaceae.

Notably, a total of 208 DSE strains were isolated from the root systems of both plants (Table 2). Moreover, 110 strains and 18 species were isolated from *S. involucrata*, while 98 strains and 22 species were isolated from *R. crenulata*. These strains belonged to 4 classes, 6 orders, 12 families, 16 genera, and 31 species. Among the 16 genera of DSE, 8 genera were shared by both plants, including *Phoma*, *Alpinaria*, *Alternaria*, *Rhexocercosporidium*, *Leptodophora*, *Leptosphaeria*, *Cadophora*, *Neonectria*, etc. The DSE genera unique to *S. involucrata* include *Cladosporium*, *Exophiala*, and *Crocicreas*. The DSE genera unique to *R. crenulata* include *Paraphoma*, *Cyphellophora*, *Leptodontidium*, *Phialocephala*, *Botrytis*, and *Microdochium*.

### 3.2. Analysis of the Community Composition and Diversity of Endophytic Fungi in Two Alpine Medicinal Plants from Different Sampling Sites

The Venn diagram analysis of culturable endophytic fungal communities in two medicinal plants revealed distinct fungal compositions across sampling sites. Ten fungi were exclusive to XW, including *Cladosporium* sp., *C. delicatulum, Neosetophoma cerealis, Penicillium fellutanum, Exophiala* sp., *Rhexocercosporidium carotae, Tetracladium maxilliforme, L. sclerotioides, Cadophora* sp., and *Neonectria* sp. Seven species were unique to XH, such as *Calyptella* sp., *Mycena citrinomarginata, Alternaria tenuissima, Dactylaria* sp., *Leptodophora gamsii, Cadophora ferruginea*, and *Fusarium* sp. Five species, including *Rhizoctonia* sp., *Alternaria sorghi, Clonostachys rosea, Trichoderma polysporum, and Neonectria lugdunensis*, were specific to XDN. XBL harbored two endemic fungi, *Alternaria longipes* and *Crocicreas* sp. A total of 22 fungi were specific to HT, including *Pezicula melanigena, Porostereum spadiceum, Paraphoma chrysanthemicola, P. salicis, Paraphoma* sp., *Phaeosphaeria, Alternaria chlamydosporigena, A. gansuensis, Alternaria* sp., *Curvularia nodulosa, Pleosporales* sp., *Pyrenophora fugax*, *Dothideomycetes* sp., *Cyphellophora* sp., *Rhinocladiella similis, Xanthoria resendei, Leptodophora echinate, Cadophora* cf. *interclivum, Phialemonium* cf., *Graphium penicillioides, Fusarium solani,* and *Thelonectria* sp. Seven fungi were unique to HW, including *Sporidesmium spiraeae*, Helotiales sp., *Leptodontidium* sp., *Phialocephala* sp., *Microdochium* sp., *Fusarium venenatum*, and *Acremonium sclerotigenum*. Five species, such as *Penicillium camemberti, Penicillium glabrum*, *Leptodontidium, Ogataea naganishii,* and *Mortierella* sp., were exclusive to HBL. Finally, two species, *Alternaria doliconidium* and *F. oxysporum*, were endemic to HDN (Figure 2C).

A total of 47 species of endophytic fungi were isolated from the roots of *S. involucrata* across four sampling sites: XW, XBL, XDN, and XH, with 21, 11, 11, and 16 species identified at each site, respectively. The fungal richness was highest at the XW site. The highest similarity (0.25) was observed between XW and XBL, with shared species including *P. meliloti, Cistella* sp., *L. orchidicola*, and *Rhexocercosporidium* sp. In contrast, the lowest similarity (0.0625) occurred between XW and XDN, where *Rhexocercosporidium* sp. was the only shared species (Table 3).

A total of 64 endophytic fungal species were isolated from the roots of *R. crenulata* across the 4 sampling sites HW, HBL, HDN, and HT. Among them, 26 species were isolated from HW, 11 from HBL, 5 from HDN, and 36 from HT. The highest fungal diversity was observed at the HT site, while the lowest diversity was found at the HDN site. The similarity between HW and HBL was the highest, with a similarity index of 0.2162, including *Cadophora spadices*, *Botrytis cinerea*, *Seaverinia geranii*, and *F. acuminatum*. The similarity between HDN and HBL was the lowest, with a similarity index of 0, indicating there were not any shared fungal species.

Statistical analysis of the abundance and diversity of endophytic fungi isolated from the eight sampling sites was conducted. It revealed that, except for the HDN site, *R. crenulata* in the HW, HBL, and HT sites exhibited a higher richness, Shannon–Wiener index, and Simpson index than *S. involucrata* at the XW, XBL, and XH sites. This suggests that *R. crenulata* has higher endophytic fungal diversity than *S. involucrata* at these locations. The Pielou index varied across sites for both plant species, but it was consistently above 0.75, indicating high evenness. Furthermore, XDN and HDN exhibited the highest values for both *S. involucrata* and *R. crenulata*, while XW and HW had the lowest values. Simultaneously, the richness and Simpson index were the lowest at XDN and HDN. These results indicate that the endophytic fungal community structures of the two plant species vary across different sites, with both *S. involucrata* and *R. crenulata* harboring rich endophytic fungal species across their respective sampling locations (Figure 2D).

### 3.3. The Dominant DSE in the Culturable Endophytic Fungal Communities of Alpine Medicinal Plants

**Figure 3:**
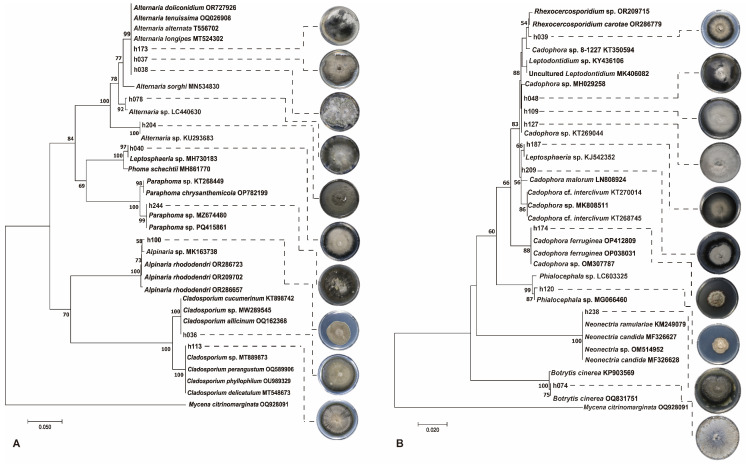
Phylogenetic tree and colony morphology of typical DSE isolated in our study. (**A**) phylogenetic tree of Dothideomycetes, (**B**) phylogenetic tree of Leotiomycetes and Sordariomycetes. The phylogenetic tree was generated in MEGA 7 using the neighbor joining method, and the robustness of the tree was evaluated using bootstrapping with 1000 replicates. On the right of the figure are the corresponding colony morphologies of DSE.

DSE represents the majority of culturable endophytic fungi in the two medicinal plants, with 208 DSE isolates accounting for 50.98% of the total culturable endophytic fungi. These include 31 species of DSE, exhibiting high diversity. Based on the morphological characteristics of the isolated DSE fungi, they were classified into 20 morphotypes (Table 4). The colonies of the isolated DSE fungi are mostly round or elliptical, with a woolly or felt-like appearance. The predominant colors are black, dark green, and gray. The edges of the colonies are relatively smooth, although a few exhibit wavy, radial, or irregular shapes. A few fungi produce red pigments in the culture medium. Among them, representative DSE fungal characteristics are shown in Figure 3.

Some common DSE fungi, such as *P. schachtii, A. rhododendri, A. alternata, Alternaria* sp., *Rhexocercosporidium* sp., *L. orchidicola, Leptosphaeria* sp., *Cadophora* sp., *N. candida*, etc., have colonized both of these alpine plants.

Rare DSE were also isolated, including some species reported infrequently in the literature, such as *A. doliconidium*, from which only a single isolate was obtained from *R. crenulata*, and *A. sorghi*, isolated as a single strain from *S. involucrata*. Other infrequently reported fungi isolated from *R. crenulata* include *Cyphellophora* sp., *Phialocephala* sp., and *Cadophora* cf. *interclivum*, while isolates from *S. involucrata* include *A. tenuissima*, *Crocicreas* sp., and *C. ferruginea,* each represented by only one isolate.

### 3.4. DSE Community Composition of Two Alpine Medicinal Plants in Different Sites

**Figure 4:**
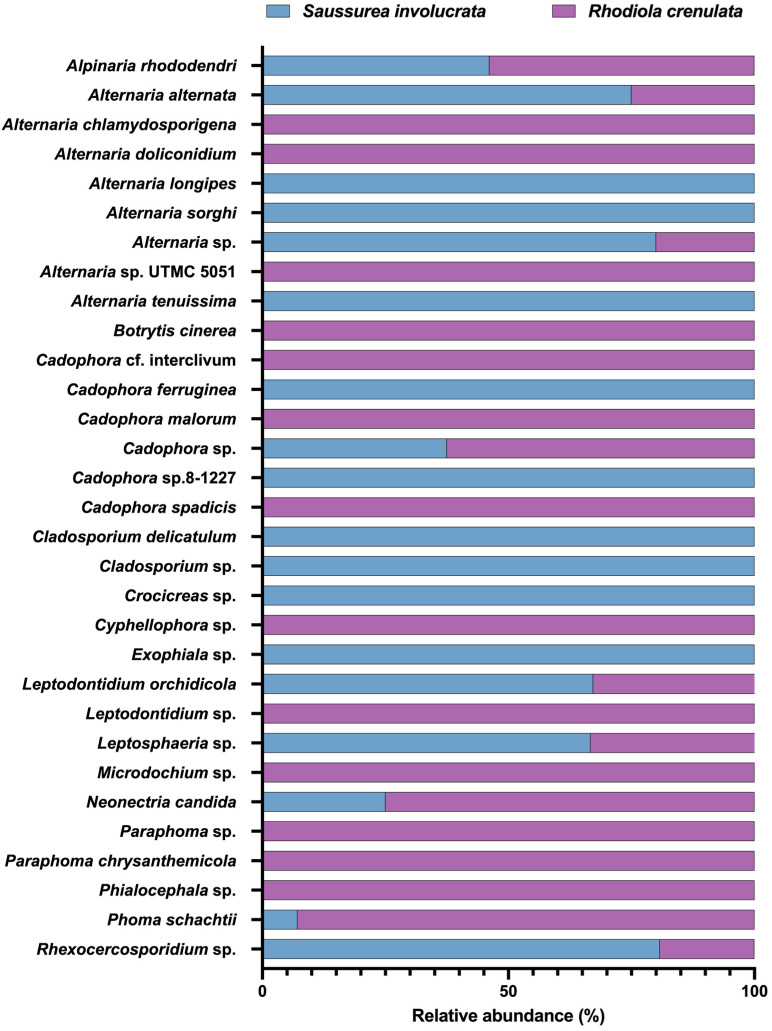
DSE community structure of two alpine medicinal plants. The figure shows the community composition and proportion of the DSE in the two alpine medicinal plants.

Analysis of the DSE community composition of 208 isolates from the two medicinal plants showed that all isolates were Ascomycota fungi, classified into 4 classes, 6 orders, 12 families, 16 genera, and 31 species. The DSE community structure exhibited some differences between the two medicinal plants (Figure 4), with 110 isolates of 18 DSE species from *S. involucrata* and 98 isolates of 22 DSE species from *R. crenulata.* Species common to both plant roots included *P. schachtii*, *A. rhododendri*, *A. alternata*, *Alternaria* sp., *Rhexocercosporidium* sp., *L. orchidicola*, *Leptosphaeria* sp., *Cadophora* sp., and *N. candida*, which were shared DSE fungi. *L. orchidicola* was the dominant species in the DSE communities of both plants, with relative isolation frequencies of 35.45% and 19.39% in *S. involucrata* and *R. crenulata*, respectively.

The host-specific DSE groups showed the distinct differences between the two plants, with 9 DSE species unique to *S. involucrata*, including *Cladosporium* sp., *C. delicatulum, A. longipes*, *A. sorghi*, *A. tenuissima*, *Exophiala* sp., *Crocicreas* sp., *C. ferruginea*, and *Cadophora* sp. Thirteen species were unique to *R. crenulata*, including *P. chrysanthemicola*, *Paraphoma* sp., *A. chlamydosporigena*, A. *doliconidium*, *Alternaria* sp., *Cyphellophora* sp., *Leptodontidium* sp., *Phialocephala* sp., *Cadophora* cf. *interclivum*, *C. malorum*, *C. spadices*, *B. cinerea*, and *Microdochium* sp. The similarity of DSE communities between the two plants was high, at 45.00%.

### 3.5. Analysis of DSE Diversity of the Two Species in Different Ways

**Figure 5:**
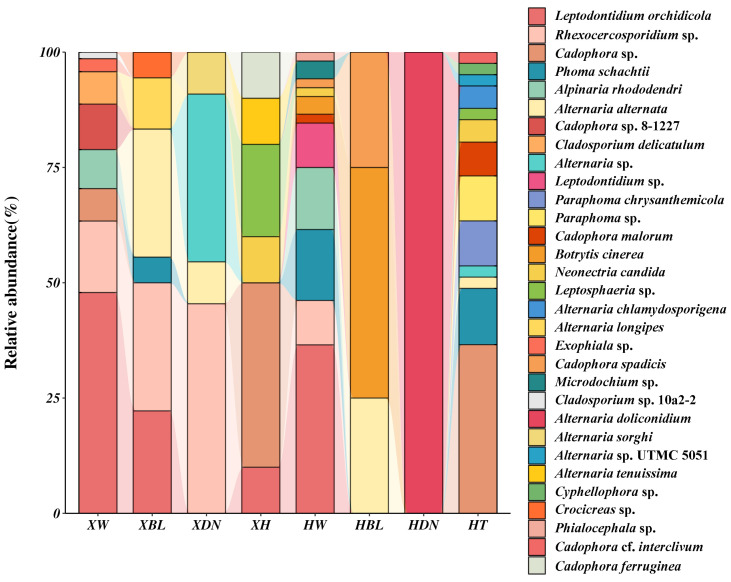
Distribution of culturable DSE in two alpine medicinal plants. The community structure of DSE in the two alpine medicinal plants varied at different sites. XW, XBL, XDN, and XH were the sampling sites of *S. involucrata,* and HW, HBL, HDN, and HT were the sampling sites of *R. crenulata*.

A statistical analysis of DSE abundance and diversity in the two plant species across different sites (Table 5) indicated that the Shannon–Wiener index in site XW was lower than in HW, whereas both XBL and XDN were higher than HBL and HDN. Only one DSE isolate was obtained from HDN, identified as *A. doliconidium*. *A. alternata* and *Rhexocercosporidium* sp. were the most widely distributed, found across four sites, followed by *L. orchidicola*, *P. schachtii*, *Cadophora* sp., and *N. candida*, which were found in three sites. Except for HDN, the evenness across the other sites was consistently high. For *S. involucrata*, diversity was highest in XW and lowest in XDN, while for *R. crenulata*, it was highest in HT and lowest in HDN. Except for HDN, the diversity indices among the sites located in the Tianshan Mountains (XW, XBL, XDN, XH, HW, and HBL) were relatively similar, whereas the HT site in the Karakoram Mountains displayed significantly higher diversity and marked differences in community composition (Figure 5). These findings suggest that the DSE community structure of the two alpine medicinal plants varies between mountain ranges.

## 4. Discussion

### 4.1. Common Characteristics of the Culturable Endophytic Fungal Communities in the Roots of Both Plant Species

This study conducted molecular identification on the endophytic fungi from the roots of two alpine medicinal plants, belonging to 3 phyla, 9 classes, 16 orders, 31 families, 54 genera, and 91 species. Among them, 20 fungal species were found in both plants, including *P. schachtii*, *P. meliloti*, *A. rhododendri*, *A. alternata*, *Alternaria* sp., *Articulospora*, *Filosporella* sp., *L. orchidicola*, *Rhexocercosporidium* sp., *Tetracladium* sp., *Leptosphaeria* sp., *Cadophora* sp., *Mycochaetophora*, Helotiales sp., *G. basitruncatum*, *D. macrodidyma*, *N. candida*, *Neonectria* sp., *T. cornearis*, and *M. arctica.* Some species, such as *Articulospora*, *Filosporella* sp., and *Tetracladium* sp., are aquatic fungi, while others like *Tetracladium* sp., *D. macrodidyma*, *M. arctica*, and *C. ferruginea* are adapted to alpine environments [32,33,34,35,36,37,38]. These fungi may help plants in high-altitude, low-temperature habitats resist cold stress, enhancing their environmental adaptability.

In the Tianshan Mountain No.1 glacier (XW and HW), Bayinbuluke, Hejing County (XBL and HBL), and Danangou Uzbek Township (XDN and HDN), both plant species were collected. Shared fungal families include Melanommataceae, a common plant endophytic fungal family [39]. Members of Didymellaceae are widely distributed across ecosystems, containing important plant pathogens and species associated with endophytic, saprotrophic, and clinical conditions [40]. Helotiaceae is a dominant endophytic family in trees, while Ploettnerulaceae is found in the marine glaciers of the Qinghai-Tibet Plateau, showing high diversity [33,41]. These fungi are frequently associated with plant tissues, promoting growth and offering enzymatic resistance to cold temperatures, helping plants survive in harsh environments. Notably, although fungal species composition varies, their functional roles often converge, providing functional redundancy that stabilizes ecosystems despite potential fungal losses due to climate change [42]. For example, isolated species such as *Cadophora* sp. and *L. orchidicola* promote plant growth, while *C. delicatulum*, *Rhexocercosporidium* sp., and *Cadophora* sp. offer disease resistance [43,44,45,46]. Collectively, these functionally analogous fungi enhance plant health and environmental stability.

### 4.2. Effects of Plant Species on Endophytic Fungal Community

Although *S. involucrata* and *R. crenulata* often coexist, the species’ differences in host plants lead to variations in the composition of their root endophytic fungal communities. In the culturable endophytic fungi of the root systems of *S. involucrata*, the dominant species was *L. orchidicola*, with an isolation frequency of 20.42%, whereas the dominant fungus in the root system of *R. crenulata* was *Filosporella* sp., with an isolation frequency of 9.68%. *L. orchidicola* was isolated from *Calypso bulbosa* and is found not only in terrestrial plant roots but also in submersed plant roots [47,48]. Studies have reported that inoculation with this fungus can increase the biomass of tomato seedlings and reduce the negative impact of pathogens on plants [43]. The genus *Filosporella,* which includes six species (*F. annelidica*, *F. aquatica*, *F. exilis*, *F. fistucella*, *F. pinguis*, and *F. versimorpha*), comprises aquatic fungi that contribute to the decomposition of fallen leaves and other organic matter in rivers [49]. These fungi are adapted to low temperatures through the production of cold-resistant enzymes [32]. Certain fungal families were specific to one plant species. For example, Ceratobasidiaceae, Cladosporiaceae, Phaeosphaeriaceae, and Mucoraceae were isolated solely from *S. involucrata*. Ceratobasidiaceae, common orchid mycorrhizal fungi (OMF) [50], aid nutrient uptake and transfer carbohydrates to the soil through symbiotic relationships with orchid plants [51]. Cladosporiaceae, found in marine environments [52], produce diverse metabolites with bioactivity and hold potential for industrial and biotechnological applications, but are also associated with plant and health hazards [53,54,55]. Fungal families such as Hydnaceae, Discinellaceae, and Mollisiaceae, found exclusively in *R. crenulata*, are common saprobes on decaying plant material in temperate regions [56]. Sclerotiniaceae include pathogenic fungi such as *Sclerotinia sclerotiorum* and *B. cinerea*, as well as the psychrophilic *S. borealis* [57]. Amphisphaeriaceae members, common plant endophytes globally, secrete bioactive secondary metabolites, including chromones, spiroketals, polyketides, terpenoids, and coumarins [58]. Mortierellaceae is among the most abundant, diverse, and widely distributed soil fungi, especially in alpine and subalpine zones [59].

Overall, *S. involucrata* and *R. crenulata* host seven and eight unique fungal families, respectively, with distinct endophytic fungal community compositions indicating that different host plants exhibit varying endophytic fungal communities, even in similar habitats. This variation may be influenced by the host plant species and their exudates. Root exudates from onions release chemical signals that alter rhizosphere microbiome recruitment [60]. Studies indicated that plants selectively “recruit” specific rhizosphere microbes [61]. Additionally, according to the “Stress Gradient Hypothesis”, interspecies cooperation increases with environmental stress [62,63]. In the harsh alpine environment, beneficial microbes may migrate from non-rhizosphere soil to the plant rhizosphere, and are then recruited into plant roots to form symbiotic relationships that aid growth and stress resistance [64,65].

### 4.3. Effects of Sampling Sites on Endophytic Fungal Community

Our findings demonstrate that the culturable endophytic fungal community structure varies across sampling sites for the same host plant. Multiple factors shape the composition of these fungal communities, including abiotic factors such as soil physicochemical properties, pH, temperature, and precipitation [66,67,68], as well as biotic factors like habitat plant composition, plant developmental stages, rhizosphere bacterial communities, and arbuscular mycorrhizal fungi (AMF) colonization [69,70,71,72]. It has been suggested that climate and spatial factors are the primary drivers of endophytic fungal community structure, with a greater impact than soil characteristics, host genotype, or geographic distance [73]. Additional studies proposed that various endophytic fungi exhibit distinct niche preferences [67]. These findings supported the concept that different fungal lineages occupy specific ecological niches, indicating functional complementarity within endophytic fungal communities. Due to the interactions among the host identity, environmental factors, and microbial communities, distinct fungal taxa may adopt unique strategies depending on environmental context [74]. Among the five sampling sites in our study (XW and HW, XBL and HBL, XDN and HDN, XH, and HT), three of them (XW and HW, XBL and HBL, XDN and HDN, and XH) are located in the Tianshan Mountains, while HT is situated in the Karakoram Mountains. Differences in vegetation cover and climate factors across mountain ranges may contribute to variations in endophytic fungal communities within the same species in different regions [75,76]. Additionally, traditional isolation methods for endophytic fungi may not capture certain unculturable endophytic fungi, and the isolation process itself may involve certain stochastic elements, which could also contribute to variations in culturable endophytic fungal community composition.

### 4.4. Potential Ecological Roles of Culturable DSE

In our study, DSE accounted for 50.98% of all culturable endophytic fungi, indicating that DSE fungi represent a significant component of the endophytic fungal communities in alpine medicinal plants. This finding aligns with previous research on the endophytic fungal communities in alpine plants [77]. The primary identified DSE fungi were classified into 4 classes, 6 orders, 12 families, 16 genera, and 31 species, with high species diversity. These fungi also exhibited high functional diversity, with *A. sorghi* and *P. chrysanthemicola* showing antioxidant properties, *Leptosphaeria* sp. and *Leptosphaeria* sp. alleviating water stress, and species like *C. delicatulum*, *Cladosporium* sp., *Paraphoma* sp., *Rhexocercosporidium* sp., and *Cadophora* sp. displaying antimicrobial activity [78,79,80,81,82]. Other species, such as *A. tenuissima*, demonstrated the capacity to produce secondary metabolites like flavonoids [46]. Additionally, *L. orchidicola* and *Microdochium* sp. were found to promote plant growth [43,83].

DSE fungi are widespread across nearly all ecosystems, colonizing inside plant roots [84]. Their high tolerance to heavy metals, drought, and salinity, particularly in extreme environments such as alpine regions, subarctic zones, deserts, wetlands, saline–alkaline soils, and areas with heavy metal contamination, highlights their essential ecological role [22,23,85,86,87,88]. In this study, both alpine medicinal plants were found to share multiple DSE species, with 9 species identified in both plants. These shared DSE fungi include species common to extreme environments, such as *Alternaria* sp., *A. alternata, L. orchidicola*, and *Leptosphaeria* sp., which are frequently found in alpine, desert, and deep-sea habitats [43,47,78,89]. Certain DSE fungi, such as *Cadophora* sp., are associated with heavy metal-contaminated soils, while others, like *Rhexocercosporidium* sp. and *Leptosphaeria* sp., colonize medicinal plants. Additionally, some fungi present in both plants, such as *Rhexocercosporidium* sp., and *N. candida*, are potential pathogens [45,90,91,92]. According to the “habitat adaptation hypothesis,” plants may enhance their resistance to harsh environments through symbiosis with endophytic fungi, which can confer similar stress tolerance to phylogenetically distant plants [22,93]. The shared DSE species may aid host plant adaptation to extreme alpine environments by alleviating environmental stress, deterring pests, and providing other resistance mechanisms. This study also revealed that certain fungi co-occurred in the roots of both plants at specific sites, such as *A. rhododendri* at site XW and HW, and *A. alternata* at site XBL and HBL. *A. rhododendri* has rarely been reported and is identified here for the first time as a DSE in alpine medicinal plants. Notably, functional similarity extends beyond shared DSE species to include distinct DSE fungi in each plant with similar functions. For example, *Cladosporium* sp., isolated only from *S. involucrata*, and *Paraphoma* sp., isolated only from *R. crenulata*, both exhibit antibacterial properties. *A. sorghi*, found exclusively in *S. involucrata*, and *P. chrysanthemicola*, isolated solely from *R. crenulata*, both possess antioxidant properties [78,94,95,96].

The DSE community composition differs between the two medicinal plants, with the DSE diversity of *R. crenulata* being higher than that of *S. involucrata*, potentially influenced by the host plant. Additionally, a diverse DSE community may aid *R. crenulata* in better adapting to extreme alpine environments, which could explain its broader distribution compared to *S. involucrata*. Apart from the aforementioned influencing factors, variations in DSE community composition across geographical locations may also relate to stress conditions specific to each site. Our isolated DSE communities exhibit functional diversity as well, including species such as *Paraphoma* sp., *P. chrysanthemicola*, *Alternaria* sp., *Leptosphaeria* sp., *Exophiala* sp., and *L. orchidicola*, commonly found in arid regions and associated with alleviating water stress and promoting the growth of desert plants [48,78,81,97,98,99]. Similarly, in extreme environments like alpine, polar, and marine habitats, where plants endure low temperatures and oxygen, species like *C. delicatulum*, *A. alternata*, *Alternaria* sp., *Cyphellophora* sp., *Phialocephala* sp., *C. ferruginea,* and *C. malorum* may help plants withstand these stresses [33,45,89,98,100,101,102]. In environments contaminated by heavy metals, species like *A. chlamydosporigena*, *Cadophora* sp., and *Microdochium* sp. may help alleviate heavy metal stress in soils [44,103]. Thus, the distinct DSE communities in these two medicinal plants might partly explain their differing distributions. The wider distribution of *R. crenulata* compared to *S. involucrata* may be attributed not only to the inherent characteristics of the plant but also to the distinct functions of its endophytic DSE fungi. For instance, *P. chrysanthemicola*, unique to *R. crenulata*, improves the rhizosphere environment, while *A. chlamydosporigena* displays heavy metal tolerance [103]. Similarly, *A. sorghi* and *A. tenuissima*, present only in *S. involucrata*, possess antioxidant capabilities [27,46].

It is noteworthy that *A. chlamydosporigena*, *A. doliconidium*, *A. sorghi*, *Crocicreas* sp., *L. orchidicola, Leptosphaeria* sp., *Phialocephala* sp., *C. spadices*, *N. candida*, *P. schachtii*, and *A. rhododendri*, among the isolated DSE fungi in this experiment, are reported for the first time in alpine environments.

In addition, *Cyphellophora* sp., *P. schachtii*, *A. rhododendri*, *L. orchidicol*, *A. chlamydosporigena*, *A. longipes*, *Crocicreas* sp., *C. ferruginea*, *C. spadices*, and *N. candida* are reported for the first time as DSE fungi in medicinal plants.

In this study, we isolated multiple fungal strains from the roots of the alpine medicinal plants. Although limited by sample size and unable to capture the entire microbial community, our findings undeniably contribute to the diversity of microbial resources in this region. A wider range of isolation techniques (such as low-temperature and oligotrophic conditions) and culture conditions (such as varied light exposure, temperature, and nutrient availability) should be explored in future studies to obtain additional culturable strains. This experiment provides valuable fungal strains for future research, which could be selected for application in low-altitude cultivation. Moreover, future studies could focus on screening strains capable of producing secondary metabolites to enhance the quality and medicinal value of herbal materials, laying a foundation for improved cultivation and quality of medicinal plants.

## 5. Conclusions

Our study showed that the composition of the culturable endophytic fungal community in two alpine medicinal plants varies according to host plants and sampling sites. Among them, the dominant fungal phylum was Ascomycota. *Leptodontidium* and *Filosporella* were the predominant genera in *S. involucrata* and *R. crenulata*, respectively. *L. orchidicola* dominated the Tianshan Mountain No.1 glacier (XW and HW), while *A. alternata* and *Rhexocercosporidium* sp. were prevalent in the Bayinbuluke (XBL and HBL). *Rhexocercosporidium* sp. was dominant in the Danangou Uzbek Township (XDN and HDN), and *Cadophora* sp. was common in both the Houxia (XH) and Taxkorgan (HT) plots. DSE fungi were the primary group in the endophytic fungal communities of these plants. *R. crenulata* exhibited a higher diversity of culturable endophytic fungal communities than *S. involucrata*, with 20 fungal species shared between the two plant species. The community structure of endophytic fungi differed between the two plant species and across sampling sites. Among the culturable endophytic fungal communities in different sites of *S. involucrata*, the XW plot located in the Tianshan Mountains was the most abundant, while the HT plot, located in the Kunlun Mountains, showed the highest diversity for *R. crenulata*. This suggests that host plants may selectively recruit endophytic fungi and that the environment may play a role in driving endophytic fungal community composition. In addition, there were both similarities and differences in the community structure of DSE. *L. orchidicola* was the dominant species in the DSE communities of both plants. Apart from the 9 DSE species shared by both plants, 9 DSE species were unique to *S. involucrata,* and 13 DSE species were unique to *R. crenulata*. Understanding the diversity of endophytic fungi in *S. involucrata* and *R. crenulata* not only provides a research basis and endophytic fungal resources for revealing their potential functions in alpine medicinal plants but also contributes to the low-altitude and high-quality artificial cultivation of these two medicinal plants in the future. The current results are mainly derived from the data of culturable endophytic fungi, and their abundance needs to be further verified by high-throughput sequencing of larger sample sizes. Moreover, to determine the taxonomy of the DSE strains from our study, multi molecular genetic markers are being used to find potential new taxa. In the next step, we will screen strains that can produce secondary metabolites to improve the quality of medicinal materials and provide a basis for the low-altitude cultivation of alpine medicinal plants.

## Figures and Tables

**Table 1:** Information on the collection of *S. involucrata* and *R. crenulata* in our study.

Mountains	Geographical Symbol	Host Plant’s Species and Numbers	Sampling Site	Geographical Location	Altitude
Tianshan Mountains	XW	6 *S. involucrata*	Tianshan Mountain No.1 glacier	42°59′23″ N 86°24′7″ E	3259.2
	HW	11 *R. crenulata*	Tianshan Mountain No.1 glacier	42°59′23″ N 86°24′7″ E	3259.2
	XBL	6 *S. involucrata*	Bayinbuluke, Hejing County	43°4′5″ N 86°44′9″ E	3502.1
	HBL	12 *R. crenulata*	Bayinbuluke, Hejing County	43°4′5″ N 86°44′9″ E	3502.1
	XDN	14 *S. involucrata*	Danangou Uzbek Township	43°31′6″ N 90°17′19″ E	3236.3
	HDN	3 *R. crenulata*	Danangou Uzbek Township	43°31′6″ N 90°17′19″ E	3236.3
	XH	8 *S. involucrata*	Houxia, Urumqi	43°9′51″ N 87°11′18″ E	3317.1
Kunlun Mountains	HT	12 *R. crenulata*	Taxkorgan	42°35′24″ N 86°32′47″ E	3374.4

**Table 2:** DSE fungi isolated from two alpine medicinal plants.

Phylum	Class	Order	Family	Genus	Species
Ascomycota	Dothideomycetes	Capnodiales	Cladosporiaceae	*Cladosporium*	*Cladosporium* sp.
					*Cladosporium delicatulum*
		Pleosporales	Didymellaceae	*Phoma*	*Phoma schachtii*
			Incertae sedis, Pleosporales	*Paraphoma*	*Paraphoma chrysanthemicola*
					*Paraphoma* sp.
			Melanommataceae	*Alpinaria*	*Alpinaria rhododendri*
			Pleosporaceae	*Alternaria*	*Alternaria alternata*
					*Alternaria chlamydosporigena*
					*Alternaria doliconidium*
					*Alternaria longipes*
					*Alternaria sorghi*
					*Alternaria* sp.
					*Alternaria* sp. UTMC 5051
					*Alternaria tenuissima*
	Eurotiomycetes	Chaetothyriales	Cyphellophoraceae	*Cyphellophora*	*Cyphellophora* sp.
			Herpotrichiellaceae	*Exophiala*	*Exophiala* sp.
	Leotiomycetes	Helotiales	Incertae sedis, Helotiales	*Crocicreas*	*Crocicreas* sp.
				*Rhexocercosporidium*	*Rhexocercosporidium* sp.
			Leptodontidiaceae	*Leptodontidium*	*Leptodontidium orchidicola*
					*Leptodontidium* sp.
				*Leptosphaeria*	*Leptosphaeria* sp.
			Mollisiaceae	*Phialocephala*	*Phialocephala* sp.
			Ploettnerulaceae	*Cadophora*	*Cadophora* cf. *interclivum*
					*Cadophora ferruginea*
					*Cadophora malorum*
					*Cadophora* sp.
					*Cadophora spadicis*
					*Cadophora* sp. 8-1227
			Sclerotiniaceae	*Botrytis*	*Botrytis cinerea*
	Sordariomycetes	Amphisphaeriales	Amphisphaeriaceae	*Microdochium*	*Microdochium* sp.
		Hypocreales	Nectriceae	*Neonectria*	*Neonectria candida*

**Table 3:** Diversity and similarity of culturable endophytic fungal communities in roots of two alpine medicinal plants in different sampling sites.

Sample Sites	Sorenson’s Similarity Coefficient								Diversity Index			
	XW	XBL	XDN	XH	HW	HBL	HDN	HT	Richness	Shannon–Wiener	Simpson	Pielou
XW	1.0000								21.0000	2.3922	0.8600	0.7857
XBL	0.2500	1.0000							11.0000	2.1609	0.8611	0.9012
XDN	0.0625	0.1818	1.0000						11.0000	2.1775	0.8600	0.9081
XH	0.1622	0.0741	0.0741	1.0000					16.0000	2.4992	0.8878	0.9014
HW	0.2979	0.3243	0.0541	0.1429	1.0000				26.0000	2.7477	0.9142	0.8433
HBL	0.0625	0.0909	0.0909	0.0000	0.2162	1.0000			11.0000	2.3035	0.8889	0.9606
HDN	0.0769	0.0000	0.0000	0.0000	0.0645	0.0000	1.0000		5.0000	1.5607	0.7778	0.9697
HT	0.1404	0.0851	0.1277	0.1923	0.1935	0.0426	0.0976	1.0000	36.0000	3.1403	0.9217	0.8763

**Table 4:** Colony characteristics of the 20 DSE morphotypes and corresponding locations.

Type of Morphology	Corresponding Lications	Fungal Species	No. of Strains	HOST Plants	Color of Colonies	Characteristics of Colonies
Type 1	XW	*Cladosporium* sp.	h036	*S. involucrata*	Olive green	The colony was loose and felt-like in texture, with a surface covered in villi. Some hyphae were basal, and margins were radial.
Type 2	XH, XDN, HT	*A. alternata*	h038	*S. involucrata*, *R. crenulata*	Gray	The colony was dense and villous, with well-developed aerial structures and irregular margins.
Type 3	XW, XH, HT	*Cadophora* sp.	h048	*S. involucrata*, *R. crenulata*	Olive green, middle gray	The colony was loose with a felt-like surface, covered in villi, partially basal, with a radial margin and a slight central elevation.
Type 4	XDN, HT	*Alternaria* sp.	h079	*S. involucrata*, *R. crenulata*	Gray	The colony was loose, felt-like, covered with white villi on the surface, with irregular margins.
Type 5	HW	*P. schachtii*	h092	*S. involucrata*, *R. crenulata*	Black, middle brown	The colony was relatively loose and felt-like in texture, slightly raised in the middle, part of the basal mycelium, with radial margin.
Type 6	XW, HW	*A. rhododendri*	h100	*S. involucrata*, *R. crenulata*	Green, middle black	The colony was loose, intrabasal, with irregular wavy edges.
Type 7	XW, HW	*L. orchidicola*	h109	*S. involucrata*, *R. crenulata*	Dark green	The colony was loose, felt-like, covered with villi, and some were intrabasal hyphae with irregular margins.
Type 8	XW	*Cladosporium delicatulum*	h113	*S. involucrata*	Olive green, middle brown	The colony was loose, felt-like, with a villous surface; colonies were flat and wrinkled, with a neat, radial edge.
Type 9	HW	*Phialocephala* sp.	h120	*R. crenulata*	Brown	The colony was loose and villous, with well-developed aerial hyphae, some basal hyphae, and a neat colony edge.
Type 10	HW	*Leptodontidium* sp.	h127	*R. crenulata*	Gray	The colony was dense and felt-like, with well-developed aerial mycelium, slightly raised overall.
Type 11	HT	*Paraphoma* sp.	h244	*R. crenulata*	Olive green, middle white	The colony was loose and felt-like, with a raised white central part and irregular margins.
Type 12	HT	*A. doliconidium*	h240	*R. crenulata*	Olive green	The colony was loose and felt-like, with a predominantly intrabasal structure.
Type 13	HT	*Cadophora* cf. *interclivum*	h209	*R. crenulata*	Black, middle white	The colony was dense, covered with white villi on the surface, slightly raised in the middle, with radial margins, and some were basal hyphae.
Type 14	HT	*Alternaria* sp.	h208	*R. crenulata*	Olive green	The colony was loose, producing red pigment, felt-like, slightly raised in the middle, with irregular margins.
Type 15	XH, HT	*Leptosphaeria* sp.	h236	*S. involucrata*, *R. crenulata*	Olive green	The colony was dense, the aerial mycelium was well developed, slightly raised overall, with an irregular edge, and some were basal mycelium.
Type 16	XH, HT	*Leptosphaeria* sp.	h188	*S. involucrata*, *R. crenulata*	Dark green, middle white	The colony was loose, flat, slightly raised in the middle, primarily intrabasal with neat margins.
Type 17	XH	*Cadophora* sp.	h176	*S. involucrata*, *R. crenulata*	Olive green, middle brown	The colony was loose, felt-like, and concentrically round, covered with brown villi on the surface, raised in the middle, with neat edges and exudates.
Type 18	HW, HBL	*Cadophora spadicis*	h091	*R. crenulata*	Red brown	The colony was loose, flat, basal hyphae with red aerial hyphae in the middle, with radial margins.
Type 19	XH, HT	*Leptosphaeria* sp.	h188	*S. involucrata*, *R. crenulata*	Green	The colony was loose, felt, covered with white villi on the surface, raised in the middle, with neat edges and exudates.
Type 20	HT	*Cadophora malorum*	h230	*R. crenulata*	Dark green	The colony was loose and villous, with white surface and slightly raised brown surface and wavy margins.

**Table 5:** Diversity of DSE fungal communities in two alpine medicinal plants at different sites.

Sampling Sites	Richness Index	Shannon–Wiener Index	Simpson Index	Pielou Index
XW	8.0000	1.6130	0.7189	0.7757
XBL	6.0000	1.6112	0.7778	0.8992
XDN	4.0000	1.1622	0.6446	0.8384
XH	6.0000	1.6094	0.7600	0.8982
HW	11.0000	1.9307	0.8018	0.8052
HBL	3.0000	1.0397	0.6250	0.9464
HDN	1.0000	0.0000	0.0000	NA
HT	13.0000	2.1080	0.8186	0.8219

## Data Availability

Data are contained within the article and Supplementary Materials.

## Supplementary Materials

The following supporting information can be downloaded at: https://www.mdpi.com/article/10.3390/jof11020113/s1, Table S1: Culturable endophytic fungi isolated from two alpine medicinal plants and the number of strains in different host plants.
