# Supplementary material for: Composition and Biodiversity of Culturable Endophytic Fungi in the Roots of Alpine Medicinal Plants in Xinjiang, China
Source: J Fungi (Basel). 2025 Feb 3;11(2):113. doi: 10.3390/jof11020113 (PMC11856231; doi:10.3390/jof11020113)
Supplement: Supplementary file 1 [file jof-11-00113-s001.zip › jof-3393314-supplementary.pdf]

Table S1. Culturable endophytic fungi isolated from two alpine medicinal plants and the number of strains in different host plants.

**Phylum**	**Class**	**Order**	**Family**	**Genus**	**Species**	Number of strains	*Saussurea involucrata*	*Rhodiola crenulata*
Basidiomycota	Agaricomycetes	Cantharellales	Ceratobasidiaceae	*Rhizoctonia*	*Rhizoctonia* sp.	1	1	
			Hydnaceae	*Pezicula*	*Pezicula melanigena*	1		1
					Pezicula radicicola	3		3
		Agaricales	Incertae sedis	*Calyptella*	*Calyptella* sp.	1	1	
			Mycenaceae	*Mycena*	*Mycena citrinomarginata*	7	7	
		Polyporales	Phanerochaetaceae	*Porostereum*	*Porostereum spadiceum*	1		1
Ascomycota	Dothideomycetes	Capnodiales	Cladosporiaceae	*Cladosporium*	*Cladosporium* sp. 10a2-2	1	1	
					Cladosporium delicatulum	5	5	
		Pleosporales	Didymellaceae	*Phoma*	*Phoma schachtii*	14	1	13
			Incertae sedis, Pleosporales	*Paraphoma*	*Paraphoma chrysanthemicola*	4		4
					Paraphoma salicis	2		2
					Paraphoma sp.	4		4
				Plenodomus	*Plenodomus meliloti*	6	4	2
			Melanommataceae	*Alpinaria*	*Alpinaria rhododendri*	13	6	7
			Phaeosphaeriaceae	*Neosetophoma*	*Neosetophoma cerealis*	1	1	
				Phaeosphaeria	uncultured *Phaeosphaeria*	1		1
			Pleosporaceae	*Alternaria*	*Alternaria alternata*	8	6	2
					Alternaria chlamydosporigena	2		2
					Alternaria doliconidium	1		1
					Alternaria gansuensis	1		1
					Alternaria longipes	2	2	
					Alternaria sorghi	1	1	

				Alternaria sp.	5	4	1
				Alternaria sp. UTMC 5051	1		1
				Alternaria tenuissima	1	1	
			Curvularia	*Curvularia nodulosa*	1		1
			Pleospora	*Pleosporales sp.*	1		1
			Pyrenophora	*Pyrenophora fugax*	1		1
		/	*Sporidesmium*	*Sporidesmium spiraeae*	1		1
	/	/	/	Dothideomycetes sp. AS5-1	1		1
				Penicillium camemberti	1		1
	Eurotiales	Aspergillaceae	*Penicillium*	*Penicillium fellutanum*	1	1	
Eurotiomycetes				*Penicillium glabrum*	3		3
		Cyphellophoraceae	*Cyphellophora*	*Cyphellophora* sp.	1		1
	Chaetothyriales	Herpotrichiellaceae	*Exophiala*	*Exophiala* sp.	2	2	
			Rhinocladiella	*Rhinocladiella similis*	1		1
Lecanoromycetes	Teloschistales	Teloschistaceae	*Xanthoria*	*Xanthoria resendei*	1		1
		Discinellaceae	*Pezoloma*	Pezoloma cf. websteri	6		6
		Helotiaceae	*Articulospora*	uncultured *Articulospora*	6	1	5
		/	/	Helotiales sp. YT-2016-2	2		2
			Cistella	*Cistella* sp.	2	2	
			Crocicreas	*Crocicreas* sp.	1	1	
Leotiomycetes	Helotiales		*Dactylaria*	*Dactylaria* sp.	1	1	
		Incertae sedis, Helotiales	*Filosporella*	*Filosporella* sp.	22	1	21
			Leptodophora	*Leptodophora echinata*	1		1
				Leptodophora gamsii	1	1	
			Rhexocercosporidium	*Rhexocercosporidium carotae*	1	1	

				Rhexocercosporidium sp.	26	21	5
			Tetracladium	*Tetracladium* sp.	4	2	2
				Tetracladium maxilliforme	5	5	
			Leptodontidium	*Leptodontidium orchidicola*	58	39	19
				Leptodontidium sp.	5		5
		Leptodontidiaceae		uncultured *Leptodontidium*	1		1
			Leptosphaeria	*Leptosphaeria sclerotioides*	1	1	
				Leptosphaeria sp.	3	2	1
		Mollisiaceae	*Phialocephala*	*Phialocephala* sp.	1		1
		Ploettnerulaceae	*Cadophora*	*Cadophora* cf. interclivum	1		1
				Cadophora ferruginea	1	1	
				Cadophora malorum	4		4
				Cadophora sp.	24	9	15
				Cadophora spadicis	2		2
				Cadophora sp. 8-1227	7	7	
			Mycochaetophora	*Mycochaetophora* sp.	2	2	
				uncultured *Mycochaetophora*	35	23	12
		Sclerotiniaceae	*Botrytis*	*Botrytis cinerea*	4		4
			Seaverinia	*Seaverinia geranii*	11		11
		/	/	uncultured Helotiales	26	10	16
Saccharomycetes	Saccharomycetales	Saccharomycetaceae	*Ogataea*	*Ogataea naganishii*	1		1
Sordariomycetes	Amphisphaeriales	Amphisphaeriaceae	*Microdochium*	*Microdochium* sp.	2		2
		Bartaliniaceae	*Truncatella*	*Truncatella angustata*	2		2
	Cephalothecales	Cephalothecaceae	*Phialemonium*	*Phialemonium* cf.	1		1
	Microascales	Microascaceae	*Graphium*	*Graphium basitruncatum*	2	1	1

					Graphium penicillioides Corda	1		1
		Hypocreales	Bionectriaceae	*Clonostachys*	*Clonostachys rosea*	1	1	
			Hypocreaceae	*Trichoderma*	*Trichoderma polysporum*	1	1	
			Nectriaceae	*Dactylonectria*	*Dactylonectria macrodidyma*	2	1	1
				Fusarium	*Fusarium acuminatum*	2		2
					Fusarium solani	1		1
					Fusarium sp.	1	1	
					Fusarium tricinctum	4	4	
					Fusarium venenatum	1		1
					Fusarium oxysporum	1		1
				Neonectria	*Neonectria candida*	4	1	3
					Neonectria lugdunensis	2	2	
					Neonectria sp. 1 NC-2022	4	2	2
				Thelonectria	*Thelonectria* sp.	1		1
			Incertae sedis	*Acremonium*	*Acremonium sclerotigenum*	1		1
		/	Thyridiaceae	*Thyridium*	*Thyridium cornearis*	2	1	1
	/	/	Incertae sedis, Pezizomycotina, Ascomycota	*Monodictys*	*Monodictys arctica*	3	1	2
Mucoromycota	Mortierellomycetes	Mortierellales	Mortierellaceae	*Mortierella*	*Mortierella* sp.	1		1
	Mucoromycetes	Mucorales	Mucoraceae	*Mucor*	*Mucor hiemalis*	1	1	
